# XXVIII Sespo Congress (Spanish Society of Epidemiology And Oral Public Health) Valencia, Spain. 10-11 November 2023

**DOI:** 10.4317/jced.1122335667803

**Published:** 2024-10-15

XXVIII Sespo Congress (Spanish Society of Epidemiology And Oral Public Health) Valencia, Spain, 10 - 11 November 2023 . Abstracts of the scientific communications follow.

