# Supplementary material for: XXVIII Sespo Congress (Spanish Society of Epidemiology And Oral Public Health) Valencia, Spain. 10-11 November 2023
Source: J Clin Exp Dent. 2024 Oct 15;16(Suppl 1):S1–S41. doi: 10.4317/jced.1122335667803 (PMC11559079; doi:10.4317/jced.1122335667803)
Supplement: Related texts

# Abstract: Clinical protocol for the prevention of anterior crossbite

**Authors:** M. Adobes Martín, D. Garcovich

**Affiliations:** *Director of Master's Degree in Advanced Orthodontics, European University of Valencia, Spain. E-mail: milagros.adobes@universidadeuropea.es*

## Introduction

 Sagittal malocclusions with anterior crossbite present a complex challenge when correction is needed in adulthood, often requiring combined surgical and orthodontic solutions or advanced orthodontic approaches with the use of intraoral skeletal anchorage.

## Objectives

 To present a clinical protocol for the early correction of anterior crossbite in school-age children.

## Methodology

 After a cephalometric evaluation to rule out the presence of skeletal Class III malocclusion, the patient's centric relation is assessed. Composite tracks are then constructed to stabilize the patient’s centric occlusion and to normalize the anterior incisor relationship.

## Results

 In the analyzed patients, the sagittal incisor relationship was normalized after approximately one month, leading to the regularization of mandibular dynamics.

## Conclusions

 The progression of malocclusions with anterior crossbite can be intercepted at an early age through minimally invasive dental interventions. These interventions may include the use of removable appliances and/or the strategic placement of adhesive materials on dental surfaces to achieve effective correction.

# Abstract: Community health intervention and oral health education in Mboulembou, Senegal

**Authors:** J. Busquet i Vila, M. Aemangué Márquez, M. Casorrán Michaut, M. Figueras Cabanes, C. Michaut Ravazza, S. Torrent Goñi

**Affiliations:** *Stomatologist, Primary Care Center Montilivi-Vila-Roja (Girona, Catalonia), Catalan Institute of Health, Spain. E-mail: jbusquet.girona.ics@gencat.cat*

## Introduction

 The NGO Amics de Mboulembou has been working for 17 years in Mboulembou, a rural area in inland Senegal, implementing projects to improve nutrition, healthcare, and education. These initiatives are characterized by the empowerment of the local population, with the goal of minimizing long-term intervention from external cooperants. In 2023, an oral health project was initiated.

## Objectives

 To assess the oral health status of the school-age population in Mboulembou and to enhance their oral health self-care practices.

## Material and Methods

 With approval from local health and education authorities, a training program was implemented for students, teachers, and health professionals in Mboulembou. Oral examinations were conducted on children, treatments were prioritized, and a protocol for the implementation and maintenance of tooth brushing in schools was established.

## Results

 Training in oral health was provided to teachers and health post staff. A total of 24 tooth brushing workshops were conducted among students. 89.95% of the school population was examined, with 56% showing no experience of caries. Supervised tooth brushing was maintained until the end of the school year.

## Conclusions

 Dental caries affects 44% of the school-age population in Mboulembou. The successful implementation of tooth brushing in schools was largely due to the involvement of local merchants and teachers in Mboulembou.

# Abstract: Comprehensive oral health in users and non-users of the 60-year-old program in two Chilean communes

**Authors:** C. Cristoffanini Bonino, D. Albers Busquets, V. Zaffiri Estévez, J. Zúñiga González, T. Morales Costa

**Affiliations:** *Universidad de los Andes, Santiago, Chile. E-mail: ccristoffanini@miuandes.cl*

## Introduction

 In 2005, Chile implemented the Explicit Health Guarantees Law (GES) to safeguard care for prevalent health issues under conditions of guaranteed access, financial protection, and quality. In 2007, the Comprehensive Dental Care Guarantee for 60-year-old Adults (GES-60) was introduced to improve oral health conditions and quality of life for individuals nearing retirement, who may experience reduced ability for oral hygiene and mobility to access dental care. A 2013 study demonstrated that this program altered clinical parameters in beneficiary patients, leading to a decrease in caries lesions and a significant increase in rehabilitation using removable prostheses. In 2015, a thesis from Concepción reported that caries lesions were the most prevalent pathology at program entry, with 97.2% of patients receiving definitive restorative treatment. A 2020 cross-sectional study of primary care dentists revealed that 71% had adequate knowledge of the Ministry of Health's Clinical Guide. In 2019, dental interns from our university conducted a study on 153 hospitalized patients. Subsequently, as part of the Universidad de los Andes Inpatient Hospitalization team, we conducted a retrospective observational study on 944 hospitalized patients, evaluating health indicators in users and non-users of this benefit.

## Objectives

 To evaluate the self-perception of oral health-related quality of life using the OHIP-7Sp survey; compare the use and frequency of brushing and fluoride toothpaste use in relation to the presence of periodontal disease (Basic Periodontal Examination), diabetes, and smoking; compare the DMFT index; verify access to and use of total and partial removable prostheses; and observe the presence of denture stomatitis in users and non-users of the GES-60 benefit.

## Material and Methods

 An observational study of non-concurrent two-year cohorts was conducted on 238 individuals (102 users and 136 non-users of GES-60) from the Ovalle Commune who turned 60 between 2007 and 2022. Two thesis students and two standardized assistants, all in their fourth and fifth years of Dentistry at the *Universidad de los Andes*, examined patients at a family health center in Ovalle between July 10-22, 2023.

## Results

 Significant differences were found in fluoride toothpaste use among diabetic patients and non-smokers who were GES-60 users. Greater access to removable prosthetic rehabilitation was observed in the user group.

## Conclusions

 No significant differences were found between users and non-users regarding self-perceived oral health-related quality of life and the DMFT index. However, improvements were noted in the absence of denture stomatitis among prosthesis wearers and in brushing habits with fluoride toothpaste in specific groups. We propose a new translation of the OHIP-7Sp survey.

# Abstract: Prevention in pediatric dentistry. Objective: Zero caries generationGutiérrez Navas AI, Ferrández Pujante A, Valverde Rubio MDP

**Authors:** A.I. Gutiérrez Navas, A. Ferrández Pujante, M.D.P. Valverde Rubio

**Affiliations:** *Dentist, UCAM, Spain. E-mail: anagn225@gmail.com*

## Introduction

 Dental caries is one of the most common diseases worldwide and currently represents a significant public health issue, necessitating early diagnosis and the prompt implementation of preventive strategies. For effective reduction, healthcare professionals and individuals in contact with children must possess the necessary knowledge to execute these measures.

## Objectives

 To evaluate how healthcare professionals, schools, and families currently contribute to the prevention of carious lesions in children and to assess possible preventive measures against dental caries that can be implemented at a multidisciplinary level.

## Material and Methods

 A literature review was conducted using the PubMed database, including publications from the last five years. The search strategy combined various keywords: Early childhood caries, Parents, Prevention, Pediatricians, and Nurses. Following the PRISMA method, a total of 22 scientific articles were selected.

## Results

 The analyzed information suggests that the professionals and individuals evaluated are currently insufficiently trained in oral preventive strategies and do not implement these measures at an early stage, either due to lack of knowledge or focus on other responsibilities.

## Conclusions

 At a multidisciplinary level, potential improvement strategies include distributing informational brochures, providing audiovisual materials, and offering conferences or training courses to professionals, who can then pass this knowledge on to families and schools. At the state level, it is recommended to implement preventive programs from birth to avoid the onset of caries, aiming to establish a Zero Caries Generation.

# Abstract: Relationship between dental caries and periodontal disease in a pregnant population: A cross-sectional study

**Authors:** C.M. Ros Melián, A.E. Pérez Rodríguez, L. Pérez Lajarín, A. Pérez Silva, A.J. Ortiz Ruiz, Y. Martínez Beneyto

**Affiliations:** *Dentist, University of Murcia, Spain. E-mail: carlarosodonto@gmail.com*

## Introduction

 Pregnancy can impact the occurrence of oral conditions. Numerous studies propose a relationship between dental caries and periodontitis, as these conditions share risk factors that cause dysbiosis in the oral microbiome.

## Objectives

 To evaluate the possible relationship between the presence of dental caries and periodontitis in pregnant women in the Region of Murcia, Spain.

## Material and Methods

 A descriptive cross-sectional observational study was conducted on pregnant women enrolled in an oral prevention program. Data collected included sociodemographic information, knowledge, caries indices (ICDAS and DMFT), CAMBRA, and periodontal indices.

## Results

 The total sample consisted of 50 pregnant women. ICDAS codes 2 and 3 were more common in patients with periodontitis, while codes 1 and 2 were more prevalent in patients without periodontitis. The mean DMFT in the periodontal and non-periodontal groups was 9.20 and 6.16, respectively. Low individual caries risk predominated in pregnant women without periodontitis, while high risk was more common in patients with periodontitis. The total BPE had a mean of 3.12 in periodontal pregnant women and 1.32 in non-periodontal pregnant women, with higher bleeding and plaque indices in periodontal patients. In pregnant women without periodontitis, 60% had gingivitis, while in pregnant women with periodontitis, stage II and grade A predominated.

## Conclusions

 There is a statistically significant association between individual caries risk, DMFT caries index, and ICDAS codes 2 and 3 with the presence of periodontitis in a pregnant population.

# Abstract: From odontogenic fistula to squamous cell carcinoma: The importance of early diagnosis. A case report

**Authors:** E. Flores Gudiño, H. Viñals Iglesias, M. Sabater Recolons, J. Godoy Flores, O. Godoy Flores, N. Flores Gudiño

**Affiliations:** *Primary Care Dentist, CAP 17 Setembre (El Prat de Llobregat, Catalonia), Institut Català de la Salut, Spain. E-mail: clinicadentalevaflores@gmail.com*

## Introduction

 Squamous cell carcinoma of the maxillary sinus is a rare condition that can cause significant aesthetic and functional issues, making early diagnosis crucial for improving patient prognosis.

## Objectives

 To highlight the importance of early diagnosis in maxillary sinus squamous cell carcinoma through a case report.

## Case Report

 We present the case of a 61-year-old male who sought urgent care at the "CAP 17 de Setembre" dental service due to facial swelling and an intraoral fistula associated with residual roots in the second quadrant. Despite initial antibiotic treatment, the patient's condition worsened, leading to multiple referrals to the maxillofacial surgery department. Initially diagnosed as an odontogenic abscess, and later as a left malar odontogenic fistula, the patient's condition continued to deteriorate. Four months after the initial referral, a computed tomography scan suggested a differential diagnosis between chronic fungal invasion and nasosinusal carcinoma. Subsequently, squamous cell nasosinusal carcinoma (cT4N2cM0) was confirmed, and the patient received induction chemotherapy concurrent with radiotherapy.

## Conclusions

 This case underscores the importance of thorough clinical examination for the early diagnosis of slow-progressing diseases that lack pathognomonic signs in their initial stages. It also highlights the crucial role of primary healthcare in cancer detection, emphasizing the need for heightened awareness and prompt action in cases of persistent or worsening oral symptoms.

# Abstract: Artificial intelligence in dentistry in Spain (2023)

**Authors:** F.D. Pérez Pastor, L. López Fuentes, M. Ortiz del Castillo, J. Jiménez Recaredo, A. Crespí Tobeña, A. Bennasar Figueras

**Affiliations:** *Primary Care Management Mallorca (IB-Salut), Spain. E-mail: nidrell@gmail.com*

## Introduction

 Artificial Intelligence (AI) is a transversal technology that enables the automation of numerous complex and/or routine tasks, including those in dentistry. Human intervention is essential in both its development and application. AI is currently in an expansion phase, and its limitations and possibilities are not yet fully understood. Although AI in dentistry lags behind its application in medicine, interest in its potential uses is growing.

## Objectives

 To present the projects that our research team has completed or is currently undertaking, and to highlight publications from other dental university teams applying AI in Spain in 2023.

## Material and Methods

 We reviewed our team's projects and conducted a literature search for AI applications in Spanish dentistry.

## Results

 Our team has developed a diagnostic imaging device using computer vision, an algorithm for predicting endodontic treatment outcomes using machine learning and initiated a study on the oral microbiome using deep learning. In Spain, there are fewer than ten dental research teams currently using AI, although interest is steadily increasing.

## Conclusions

 The use of AI in dentistry is still at an early stage in Spain compared to other countries. SESPO (Spanish Society of Epidemiology and Oral Public Health) is ideally positioned to carry out and develop AI projects due to the academic and intellectual capacity of its members, as well as the extensive potential of its databases.

# Abstract: Oral health-related quality of life among pregnant women with periodontal disease

**Authors:** A.E. Pérez Rodríguez, C.M. Ros Melian, L. Pérez Lajarín, A.J. Ortiz Ruiz, C. Serna Muñoz, Y. Martínez Beneyto

**Affiliations:** *University of Murcia, Spain. E-mail: 00elenaperez@gmail.com*

## Introduction

 Pregnancy is associated with an increased risk of periodontal disease, which can contribute to complications such as preeclampsia, preterm birth, and low birth weight. Additionally, periodontal disease leads to attachment loss, tooth mobility, and tooth loss, which can negatively impact the oral health-related quality of life (OHRQoL) of pregnant women.

## Objectives

 This study aims to evaluate the influence of the severity of periodontal disease on the OHRQoL in pregnant patients.

## Material and Methods

 A cross-sectional clinical study was conducted involving pregnant women enrolled in a Preventive Program. Participants completed sociodemographic questionnaires, belief assessments, and the Oral Health Impact Profile (OHIP-14) questionnaire. Periodontal indices, including plaque index, bleeding index, and probing depth (EPB), were recorded.

## Results

 The study included 50 pregnant women with a mean age of 32.74 years. Most participants were aware of the importance of oral hygiene, reporting that they brushed their teeth three times daily. Non-periodontal pregnant women had a plaque index of 30.88%, a bleeding index of 11.56%, and a mean EPB of 1. In contrast, pregnant women with periodontal disease exhibited a plaque index of 31%, a bleeding index of 20%, and a mean EPB of 3. The OHRQoL was notably worse in the periodontal group, with an OHIP-14 score of 12.64, indicating a significant relationship between periodontal disease and diminished OHRQoL in pregnant women.

## Conclusions

 The findings suggest that periodontal disease in pregnant women has a detrimental effect on their oral health-related quality of life.

# Abstract: Oral health survey of schoolchildren in the principality of Asturias 2018

**Authors:** J.M. Blanco González, A.I. Martínez-Viademonte Díaz-Canel, J.M. Montiel Company, J.E. Iranzo Cortés

**Affiliations:** *General Directorate of Public Health and Mental Health Care. Ministry of Health of the Principality of Asturias, Spain. E-mail: josemaria.blancogonzalez@asturias.org*

## Introduction

 In line with WHO recommendations on the periodicity of oral health epidemiological studies, Asturias conducted its first children's oral health survey in 1992, followed by a second in 2008. A decade later, the Regional Ministry of Health and the University of Oviedo, in collaboration with the University of Valencia's Department of Stomatology, sponsored a new survey.

## Objectives

 The objectives were to analyze the oral health status of Asturian schoolchildren during 2018-19, assess its evolution compared to previous studies, determine the prevalence of caries by age, sex, residence, and health area, evaluate periodontal health, and estimate the prevalence of malocclusions.

## Material and Methods

 An observational epidemiological study was conducted following the WHO's "Oral Health Surveys: Basic Methods" (3rd and 4th editions) recommendations, employing stratified, single-stage sampling and examiner calibration.

## Results

 The final sample included 2,149 schoolchildren (52% male, 48% female). Caries prevalence in 6-year-olds was 33.8% in primary dentition (20.2% active) and 2.7% in permanent dentition (1.3% active). Among students in their 1st year of Compulsory Secondary Education (ESO, 12-13 years), prevalence was 27.8% (6.3% active), and in the 3rd year of ESO (14-15 years), it was 33.9% (6.2% active). The dmft/DMFT indices were 0.97 for primary dentition (6 years), 0.04 for permanent dentition (6 years), 0.62 in 1st ESO, and 0.72 in 3rd ESO. The Restorative Index (RI) ranged from 45.4% to 88.9%, and the Significant Caries Index (SiC) ranged from 0.12 to 2.90 across age groups. Periodontal health was good in approximately 32% of ESO students. Malocclusions varied in prevalence, with 19.3% of 1st ESO and 20.7% of 3rd ESO students undergoing orthodontic treatment.

## Conclusions

 Compared to previous studies, there was an increase in caries prevalence in primary dentition but a decrease in permanent dentition, particularly in ESO students (12-15 years). The SiC decreased, and the RI increased significantly across all cohorts, indicating improved oral health and treatment access.

# Abstract: Efficacy of silver diamine fluoride in the treatment and prevention of dental caries after 9 months of follow-up in schoolchildren from Cameroon

**Authors:** D. González Alarcón, B.G. Akono Ngueda, M. Ashu Agbor, B.J. Dongmo, I. Cabello Malagón, M. Bravo

**Affiliations:** *Mindja Paul Health and Emancipation Research Centre. University of Yaoundé, Cameroon. E-mail: david.gonzalez.alarcon@gmail.com*

## Introduction

 Understanding dental caries as a global and social problem necessitates the exploration of accessible solutions that empower populations to improve their health beyond the availability of advanced technology and specialized dental services.

## Objectives

 To evaluate the efficacy of 38% silver diamine fluoride (SDF) in arresting and preventing caries lesions in a school population from Djoum and Mintom, Cameroon.

## Material and Methods

 An observational, descriptive, and prospective study was conducted with the necessary public authorizations and ethical committee approval. The study included 98 schoolchildren aged 6-12 years, analyzing a total of 986 teeth (n=986). Caries lesions were assessed using ICDAS-II criteria and DIAGNOdent technology. SDF was applied to the lesions, and a follow-up examination was performed after 9 months.

## Results

 Following the application of SDF on 455 primary teeth and 531 permanent teeth, a preventive fraction of 80.2% for primary dentition and 97.3% for permanent dentition was observed. In both groups, 93.6% of primary teeth and 99.5% of permanent teeth lesions did not progress to ICDAS-II scores 5-6. Additionally, DIAGNOdent measurements showed no significant increase in the highest vector of intense demineralization.

## Conclusions

 Stabilization was observed in ICDAS-II scores 1-4 across all three DIAGNOdent levels, with no significant progression to more severe demineralization. Less severe lesions demonstrated a better response to remineralization. SDF emerged as an effective, cost-beneficial alternative for caries management in this population.

# Abstract: Oral health in a child population of Cameroon

**Authors:** M.A.N. Ngoh Mankongo, S.G. Zouche Jumbon, D. González Alarcón, M. Ashu Agbor, B.J. Dongmo, A. Castaño Séiquer, C. Deube Gako

**Affiliations:** *University of Seville, Spain. E-mail: auroremankongo12@gmail.com*

## Introduction

 Reducing risk factors and implementing strategies based on primary health care can significantly help in preventing and controlling the most common oral diseases. Dental caries and gingival diseases display a pronounced social gradient, disproportionately affecting impoverished populations from an early age.

## Objectives

 To describe the oral health status of schoolchildren in Djoum and Mintom, Cameroon.

## Material and Methods

 A descriptive cross-sectional study was conducted over 6 months on children aged 6-12 years in primary schools in the rural areas of Djoum and Mintom. Data were collected using a standardized form. Caries diagnosis was performed using the ICDAS-II methodology. The periodontal status was assessed using the Community Periodontal Index (CPI), adapted for children under 15 years old.

## Results

 The study sample consisted of 377 schoolchildren. The prevalence of caries was 99.7%. Primary teeth (42%) were more affected than permanent teeth (38%), with a predominance of score 2 (P<0.05) and a predilection for posterior teeth. Regarding severity, the need for non-operative treatment was higher for ICDAS-1 (37.9%), ICDAS-2 (97.1%), and ICDAS-3 (48.8%) than for operative treatment: ICDAS-4 (21.2%), ICDAS-5 (10.3%), and ICDAS-6 (6.9%). The CPI results ranged from bleeding on probing (score 1: 55.2%) to the presence of calculus (score 2: 41.1%).

## Conclusions

 The oral health status of these schoolchildren was poor. These findings highlight the need for similar research in other communities to harmonize planning efforts focused on the promotion and protection of oral health in rural areas.

# Abstract: Neonatal intrinsic dental discoloration: A clinical case report

**Authors:** A. Ministral Masgrau, M. Amela Morcillo, M. Pulido Martí, J. Masdevall Galter, P. Ronda Sabaté, J. Solà Pou

**Affiliations:** *CAP Sarrià de Ter (Girona, Catalonia), Institut Català de la Salut, Spain. E-mail: agnesminis@gmail.com*

## Introduction

 Dental discolorations can be extrinsic or intrinsic, with the latter often resulting from local or systemic causes. Since color change alone is not a reliable diagnostic indicator, a detailed physical examination and comprehensive clinical history are essential for determining the etiology.

## Objectives

 To present a case of severe intrinsic dental discoloration in a pediatric patient and discuss its potential causes and management.

## Material and Methods

(description of the clinical case): We report the case of a 28-month-old girl presenting with intense green intrinsic discoloration of her four upper and four lower incisors. The patient had a history of premature birth at 25 weeks of gestation, leading to delayed tooth eruption. Her current medical conditions include epilepsy, cerebral palsy, and chronic kidney failure. A thorough review of the patient's clinical history and relevant literature was conducted to ascertain the origin of the discoloration.

## Results

 While the patient's current underlying conditions were not directly linked to the discoloration, her neonatal complications due to prematurity, cholestasis, and hepatobiliary pathology were identified as potential causes. Blood tests from the neonatal period revealed hyperbilirubinemia and elevated liver enzyme levels, coinciding with the formation and maturation stages of the primary incisors. This finding suggests the incorporation of biliverdin into the dentin, explaining the intense green color observed.

## Conclusions

 This case highlights the importance of early dental visits in childhood, emphasizes the need for accurate diagnosis of intense tooth discoloration even when immediate treatment is not required, and underscores the essential role of a thorough medical history in determining the underlying cause. Additionally, it is important to note that not all teeth may be affected with the same intensity. Informing parents about the diagnosis can be reassuring, and ongoing monitoring and preventive measures are crucial in managing such cases.

# Abstract: Effect of information sources used by dentists on the quality of antibiotic prescription in dentistry

**Authors:** A. Rodríguez Fernández, M. Mulero de Caso, M. Zapata Cachafeiro

**Affiliations:** *University of Santiago de Compostela, Spain. E-mail: almudena.rodriguez@usc.es*

## Introduction

 Bacterial resistance is a significant public health concern, exacerbated by the improper use of antibiotics. Dentists are responsible for approximately one-tenth of antibiotic prescriptions at the community level.

## Objectives

 To describe the sources of antibiotic information utilized by dentists and evaluate their association with the quality of antibiotic prescriptions.

## Material and Methods

 A cross-sectional study was conducted among dentists. Data were collected through an online, self-administered questionnaire distributed via messaging groups, social networks, and websites. The dependent variable "prescription quality" was defined based on the active ingredients dentists would prescribe to non-allergic and penicillin-allergic patients. Dentists who correctly identified both amoxicillin and clindamycin as appropriate antibiotics were classified as appropriate prescribers. The association between information sources and prescription quality was analyzed using a logistic regression model.

## Results

 The study included 878 dentists. Of these, 39.5% relied on a single information source, with the vademecum being the most commonly used. Attendance at courses [OR 0.64; 95%CI (0.47-0.88)], use of the Antimicrobial Therapy Guide [OR 0.44; 95%CI (0.23-0.87)], and information from scientific societies [OR 0.69; 95%CI (0.51-0.93)] were associated with improved quality of antibiotic prescriptions, whereas reliance on the vademecum was linked to poorer prescription quality [OR 1.39; 95%CI (1.00-1.93)].

## Conclusions

 The findings suggest that the sources of information and continuing education utilized by dentists significantly influence the quality of antibiotic prescriptions. Improving access to and reliance on evidence-based resources may enhance prescription practices and help mitigate the issue of bacterial resistance.

# Abstract: Incorporation of a new professional role in primary care teams: Deployment of dental hygienists in Catalonia

**Authors:** C. Vida Lucea, E. Casals Peidró, A.M. Enrich Pujol, E. Caula Pinsach, M.B. Enfedaque Montes, R. Ruiz Riera

**Affiliations:** *Oral Health Office, Strategic Directorate of Primary and Community Care, General Directorate of Health Planning and Research, Department of Health, Government of Catalonia, Spain. E-mail: cristinavida@gencat.cat*

## Introduction

 The primary care (PC) dental services portfolio in Catalonia encompasses restorative, preventive, and surgical activities. However, the predominance of surgical activities has limited the time allocated to primary prevention efforts. In response, the Department of Health has initiated the integration of dental hygienists into PC teams to expand the focus on preventive care.

## Objectives

 To integrate the role of dental hygienists into PC teams to enhance preventive dental care.

## Material and Methods

 A structured work plan was developed, which included the following steps: drafting a guide document detailing the required number of professionals and their deployment; preparing a functional plan outlining target groups, activities, and evaluation methods; designing a reception and support plan; monitoring the integration process through a follow-up survey; and creating a collaborative platform for training and disseminating updates.

## Results

 The relevant documents have been completed. By the end of the first year, 72.15% of the planned professionals were recruited. The follow-up survey identified training needs among other aspects. The reception and support plan received an average rating of 4.51/5 from the professionals involved. The collaborative platform is operational, with 269 registered hygienist users.

## Conclusions

 The work plan was executed as designed. Continued efforts are necessary to fully deploy the program and incorporate the targeted 370 dental hygienists.

# Abstract: Impact of dental hygienists' incorporation into primary care teams in Catalonia

**Authors:** E. Caula Pinsach, M. Figueras Cabanes, E. Mora Beneyto, C. Muñoz Olleta, M. Quintana Montero, C. Vida Lucea

**Affiliations:** *Oral Health Office, Strategic Directorate of Primary and Community Care, General Directorate of Health Planning and Research, Department of Health, Government of Catalonia, Spain. E-mail: salutbucodental.salut@gencat.cat*

## Introduction

 The reform of primary care teams in Catalonia has involved changes not only in the number of professionals but also in the integration of new roles to address emerging needs, including the addition of dental hygienists. The first hygienists were integrated in September 2022, leading to a significant impact on oral health preventive activities.

## Objectives

 To evaluate the impact of incorporating dental hygienists into primary care on the preventive activities carried out by primary care dentistry teams.

## Material and Methods

 A descriptive study was conducted, comparing the preventive activities performed by dentistry teams in the year prior to the incorporation of dental hygienists with those performed in the year following their integration.

## Results

 The number of patients receiving pit and fissure sealants nearly tripled in one year, with 47% of this increase directly attributed to the incorporation of hygienists. Similarly, topical fluoride applications increased by 141%, with hygienists contributing to 25% of this growth.

## Conclusions

 The integration of dental hygienists has significantly increased the population receiving preventive and health promotion services. This rise is due not only to the addition of hygienists but also to the heightened activity of dentists. Further assessment is needed to evaluate the impact on health outcomes and access to primary care dental consultations.

# Abstract: Introduction of silver diamine fluoride (SDF) as a therapeutic tool in primary care dentistry in Catalonia

**Authors:** M. Figueras Cabanes, E. Caula Pinsach, C. Vida Lucea, D. Frigola Castro, E. Mora Beneyto

**Affiliations:** *Strategic Directorate of Primary and Community Care, General Directorate of Health Planning and Research, Catalan Health Institute, Department of Health, Government of Catalonia, Spain. E-mail: mfigueras.girona.ics@gencat.cat*

## Introduction

 Addressing untreated dental caries lesions in vulnerable populations is a global challenge. The World Health Organization (WHO) recommends non-invasive treatments for early childhood caries and for patients with disabilities within primary care settings. In response, the Department of Health in Catalonia has prioritized the implementation of silver diamine fluoride (SDF) treatment in primary care dental consultations.

## Objectives

 To implement SDF treatment for dental caries lesions in Primary and Community Care (PCC) dental consultations in Catalonia.

## Material and Methods

 A timeline for treatment implementation was developed, focusing on the following key points: conducting a literature review of protocols and recommendations, drafting a treatment protocol, assessing training needs, calculating human and material resources based on attendance data from the Primary Care Services Information System, and disseminating information to primary care professionals, managers, and the general population.

## Results

 It is estimated that 35,677 patients will receive SDF treatment during the first year across 368 primary care teams, with patient numbers ranging from 50 to nearly 300 depending on the team. A protocol has been established, including target groups, indications, treatment guidelines, and tools for evaluating activities. Dissemination has been carried out among various groups, and a training session was conducted on November 6th.

## Conclusions

 The implementation of SDF treatment has proceeded according to the proposed timeline. Evaluating the effectiveness of this implementation during the first year is essential to verify the initial estimates and outcomes.

# Abstract: Accuracy and usefulness of DIAGNOdent in the diagnosis of dental caries: A case report

**Authors:** G. García Vicent, J.F. Martín Morales, M.D.R. Garcillán Izquierdo, M. De Feo Tolosa, A.L. Lenguas Silva

**Affiliations:** *Faculty of Dentistry, Complutense University of Madrid, Spain. E-mail: gergar01@ucm.es*

## Introduction

 The DIAGNOdent Pen 2190 is a diagnostic tool that uses a 655 nm laser to identify caries-affected areas in dental tissues. Variations in mineral content and dental porosity result in changes in fluorescence patterns.

## Objectives

 To evaluate the accuracy and usefulness of DIAGNOdent in diagnosing dental caries through a clinical case study.

## Material and Methods

 A male patient presented at the University Clinic of the Complutense University of Madrid with white spot lesions on the buccal surfaces of all maxillary teeth, except tooth 22 (healthy) and tooth 26 (significant tissue loss). Eight mandibular teeth exhibited interproximal enamel lesions without dentinal involvement. DIAGNOdent was utilized to assess the extent of demineralization. Treatment included in-office application of 5% NaF (22,600 ppm) once weekly for three weeks, complemented by a home care protocol.

## Results

 Initial DIAGNOdent readings indicated that on buccal surfaces, 53% of the areas were healthy, 28% showed early demineralization, and 19% had severe demineralization. Interproximal surfaces showed 50% healthy areas, 43% incipient demineralization, and 7% severe demineralization. After three months of treatment, buccal surfaces improved to 92% healthy areas and 8% early demineralization, while interproximal surfaces showed 64% healthy areas, 29% incipient demineralization, and 7% severe demineralization.

## Conclusions

 The DIAGNOdent Pen 2190 is a valuable supplementary diagnostic tool for detecting dental demineralization, allowing for precise monitoring of caries progression and treatment efficacy.

# Abstract: The importance of school education in oral health

**Authors:** J.F. Martin Morales, A.L. Lenguas Silva, G. García Vicent, G.D.J. Herrera Martínez, M.V. Mateos Moreno

**Affiliations:** *Complutense University of Madrid (UCM), Spain. E-mail: josefrma@ucm.es*

## Introduction

 Health education in schools is crucial for enabling students to apply acquired knowledge to their daily habits. Such educational interventions must be adapted to the students' socioeconomic and cultural contexts.

## Objectives

 To evaluate the effectiveness of oral health education interventions in schools with different socioeconomic levels.

## Material and Methods

 As part of the Community Dentistry program at UCM, extramural practices were conducted in two schools during the 2022-23 academic year: Claret School (high socioeconomic level) and Cañada Real School (very low socioeconomic level). The educational intervention included video presentations, phantom demonstrations, games, raffles, and interactive discussions. Pre- and post-intervention questionnaires were administered to assess the effectiveness of the health education program.

## Results

 Positive outcomes were observed in both schools. Notably, students at Cañada Real School exhibited high rates of onychophagia (72%) and regular use of tobacco, hookah, or cannabis (63%). Post-intervention, students from both schools increased their brushing frequency and duration while reducing between-meal sugar intake (50% reduction in Claret School and 100% in Cañada Real School).

## Conclusions

 Educational interventions are essential for all students, regardless of socioeconomic status. Regular implementation is necessary to ensure lasting awareness and habit formation. This study underscores the importance of tailoring oral health education to specific socioeconomic contexts for maximum effectiveness.

# Abstract: Clinical cases of fusion and gemination in primary dentition: Diagnosis and treatment options

**Authors:** V.M. Nunes Marinheiro, C.S. Garcete Delvalle

**Affiliations:** *Faculty of Dentistry, Complutense University of Madrid, Spain. E-mail: venunes@ucm.es*

## Introduction

 Fusion and gemination are two forms of dental anomalies where fusion results in one fewer tooth in the arch than normal, while gemination yields the correct tooth count when the geminated tooth is counted as one. Both conditions can present with caries along the division line, necessitating prompt diagnosis and treatment. Early identification is crucial to prevent complications in the development of the dentition.

## Objectives

 To present clinical cases of fusion and gemination in primary dentition and discuss their diagnosis and treatment options.

## Material and Methods

 Three cases involving children with complete primary dentition were analyzed. Case 1: A 3.5-year-old girl with fusion of teeth 7.2-7.3 presenting caries, which was restored, and gemination of tooth 8.2, where a fissure sealant was applied. Case 2: A 6.5-year-old boy with fusion of teeth 5.1-5.2 without pathological signs, monitored until exfoliation. The permanent successor tooth 1.2 exhibited a conoid shape. Case 3: A 4.5-year-old girl with fusion of teeth 5.1-5.2 and caries, which was treated.

## Results

 Each case displayed unique clinical characteristics that required tailored treatment approaches. Caries along the division line was observed in two cases, necessitating restorative interventions.

## Conclusions

 The treatment plan for fusion and gemination in primary dentition depends on the specific clinical situation. Emphasis should be placed on meticulous hygiene procedures along the tooth division line to prevent caries development. Regular monitoring is essential to manage potential complications and ensure proper development of the permanent dentition.

# Abstract: Detection of hidden sugars in the diet for dental students

**Authors:** A. Ruiz Miravet, T. Almerich Torres, J.E. Iranzo Cortés, A. Negre Barber, J.M. Montiel Company, J.M. Almerich Silla

**Affiliations:** *University of Valencia, Spain. E-mail: anna_xodos@hotmail.com*

## Introduction

 The amount of sugar is not the only factor related to dental caries; factors such as the frequency of intake and food consistency also play significant roles.

## Objectives

 To enhance dental students' knowledge using digital tools to understand and communicate to patients the importance of detecting hidden sugars in the diet as a crucial element in managing cariogenicity.

## Material and Methods

 The cariogenic potential of the diet was assessed in dental students (n=64) by detecting hidden sugars through reading and analyzing food and beverage labels using digital dietary diaries and other electronic materials. Knowledge acquisition was evaluated using a 20-question multiple-choice questionnaire (1 point for correct answers, 0 for incorrect answers), which students completed before and after the educational activities.

## Results

 Statistically significant differences (p<0.001) were observed between the questionnaire scores before (mean 12.43 points, 95% CI 11.65-13.20) and after (mean 14.98 points, 95% CI 14.13-15.85) the activities. Student satisfaction was evaluated with a survey consisting of 9 closed questions rated on a 0-5 Likert scale and one open-ended question. Closed questions received scores ranging from 3.26 to 4.82 out of 5.

## Conclusions

 The educational intervention revealed students' initial lack of knowledge about hidden free sugars in foods and beverages, which was corroborated by the pre- and post-activity questionnaire results and satisfaction survey. The intervention effectively improved dental students' understanding of hidden sugars and their impact on oral health.

# Abstract: A comparative study of three caries risk assessment methods: CAMBRA, Cariogram, and caries risk semaphore (CRS)

**Authors:** J.E. Iranzo Cortés, I. Català Benavent, J.M. Montiel Company, A. Ruiz Miravet, T. Almerich Torres, J.M. Almerich Silla

**Affiliations:** *University of Valencia, Spain. E-mail: j.enrique.iranzo@uv.es*

## Introduction

 The need to assess caries risk has led to the development of various methods, with CAMBRA, Cariogram, and Caries Risk Semaphore (CRS) being among the most commonly used.

## Objectives

 To determine the concordance among the CAMBRA, Cariogram, and CRS methods for assessing caries risk.

## Material and Methods

 This study involved 672 patients from the Dental Clinic of the University of Valencia, who attended Preventive and Community Dentistry II practices. Students assessed the caries risk of these patients using the three methods. A descriptive analysis of the sample was conducted, and concordance between methods was evaluated using the kappa coefficient.

## Results

 According to CRS, 48% of patients (n=321) were classified as high risk, 14% (n=96) as medium risk, and 38% (n=255) as low risk. The highest concordance was found between CRS and CAMBRA, with an unweighted kappa of 0.36. For risk severity assessments, the highest kappa values were also between CRS and CAMBRA: 0.46 for low risk, 0.14 for medium risk, and 0.40 for high risk.

## Conclusions

 There is considerable heterogeneity in the results obtained from different caries risk assessment methods. This underscores the need for further research into these methods and their predictive capabilities to determine the most effective approach. Standardizing caries risk assessment methods is crucial for improving clinical decision-making and patient care in preventive dentistry.

# Abstract: Community intervention for early childhood caries prevention

**Authors:** L. Figueroa Marcé, N. Bernardo Vilamitjana, Z. Bastardo López, M. Pujiula Blanch, E. Olabarrieta Zaro, B. Pérez Jiménez

**Affiliations:** *Primary Care Team of Salt (Girona, Catalonia), Spain. E-mail: lfigueroa.girona.ics@gencat.cat*

## Introduction

 The oral health program of the Catalan Public Health Agency revealed a higher prevalence of caries in Salt compared to the Girona region. Salt is an urban municipality with 32,517 inhabitants, 38% of whom are immigrants with low socioeconomic status.

## Objectives

 To prevent caries in children aged 6 months to 2 years from high-risk families and to improve oral health for the entire family.

## Material and Methods

 Patient recruitment was conducted in pediatric primary care consultations (dentistry and dental hygiene). Families with children affected by caries were considered high-risk. The intervention included an oral examination, brushing technique instruction, and dietary advice. Follow-up visits were recommended based on the caries risk assessment. Families were invited to participate in three multidisciplinary group sessions led by a nutritionist, emotional well-being specialist, and oral hygienist to improve eating habits, oral hygiene, and overall oral health. A follow-up evaluation to assess caries incidence is planned for one year later.

## Results

 To date, 44 families with a total of 74 infants have participated; 9 families engaged in the group intervention. Participation and acceptance have been positive, with an observed increase in oral health knowledge among participants.

## Conclusions

 This multidisciplinary and community-based intervention is expected to prevent early childhood caries and improve community oral health. The approach demonstrates the potential of integrating various healthcare disciplines to address oral health disparities in diverse, low-income populations.

# Abstract: Prevention methods in dental implant treatments for diabetic patients

**Authors:** G. Tesser, M. Vindel

**Affiliations:** *First Fit, Clinical Support, Madrid, Spain. E-mail: gaby.tesser@gmail.com*

## Introduction

 Dental implants are common in dentistry, but diabetes can increase the risk of postoperative complications. Understanding the special care required for diabetic patients undergoing dental implant treatment is crucial.

## Objectives

 To identify, through a literature review, the specific pre- and postoperative procedures recommended for patients receiving dental implant treatment.

## Material and Methods

 A search was conducted in PubMed using the keywords: Diabetes mellitus, Diabetes complications, Dental implants, Peri-implantitis, Glycated Hemoglobin, and Glucose variability, covering studies from 2000 to the present.

## Results

 Fifty articles were identified, of which 28 were included for full-text review. The review highlighted the importance of antibiotic prophylaxis and the use of chlorhexidine, as well as the need to control blood glucose levels before implant placement. Poor glycemic control was the most frequently cited risk factor associated with implant failure. Peri-implantitis was identified as the main complication linked to implant failure in diabetic patients.

## Conclusions

 Effective management of diabetic patients undergoing dental implant treatments requires stringent control of blood glucose levels and the use of preventive measures such as antibiotic prophylaxis and chlorhexidine. Addressing these factors can help mitigate the risk of complications and improve implant success rates.

# Abstract: The use of vapes and their risks to oral health

**Authors:** A.C. Estop Romero, A.V. Gracía García, A. Gutiérrez Gallardo, M.P. Navales Beltrán, R. Tarragó Gil

**Affiliations:** *Dr. Sofia Hernandez Montero Dental Clinic, Spain. E-mail: anaestop30@hotmail.com*

## Introduction

 Vapes are increasingly being used as an alternative to conventional cigarettes, offering appealing flavors and scents that contribute to significant addiction. However, the population remains inadequately informed about their adverse effects on both oral and systemic health, raising public health concerns.

## Objectives

 To highlight the detrimental effects of vaping on oral health and to emphasize that smoking cessation, rather than vaping, is the healthier option.

## Material and Methods

 A narrative review was conducted using PubMed (MEDLINE), SciELO databases, and the Cochrane Library. Keywords included "vaping," "smoking," "xerostomia," "systemic health," "periodontal health," and "vaping products." Inclusion criteria were: a) all methodological designs; b) publications in English and Spanish; c) publications from the last 8 years. Letters to the editor and case reports were excluded. A total of 18 articles were selected for review.

## Results

 Research indicates that flavorings such as diacetyl, pulegone, and metals added to vapes are highly toxic compounds. These substances are linked to adverse cardiovascular, respiratory, immunological, and periodontal effects, as well as xerostomia, which increases the risk of caries.

## Conclusions

 While vapes are less harmful than traditional tobacco, they still pose significant risks to oral and systemic health. Further research is needed to better understand their long-term effects.

# Abstract: Importance of education and motivation in mechanical plaque control to improve oral health in older adult patients

**Authors:** M.M. Vindel Castillo, G. Tesser

**Affiliations:** *FirstFit, Clinical Support, Madrid, Spain. E-mail: mmvindel@yahoo.com*

## Introduction

 Promoting oral hygiene self-care measures among older patients can positively impact their oral health and enhance treatment outcomes.

## Objectives

 To assess the effectiveness of education and motivation in mechanical plaque control for achieving commitment and participation in self-care among older adults.

## Material and Methods

 A literature search was conducted using Google Scholar and PubMed, with the search period limited from 2000 to the present. Keywords used included: Motivation, Education, Elderly, and Oral Hygiene.

## Results

 The search resulted in 44 articles from PubMed and 10 from Google Scholar. After excluding 6 articles from PubMed and 1 from Google Scholar, a total of 38 articles from PubMed and 9 from Google Scholar were included for review.

## Conclusions

 Cognitive-behavioral interventions are effective in motivating and educating older adult patients. A positive perception of behavioral change is associated with better compliance with instructions and self-care.

# Abstract: Analysis of oral hygiene level and gingival status in 1st and 6th grade primary school students from La Marina de Port neighborhood, Barcelona

**Authors:** M. Terrades Oliver, M. Guix Casas, J. Mellado Campoy, V. Sanchez Duque

**Affiliations:** *Catalan Institute of Health, University of Barcelona, Spain. E-mail: mterrades.bcn.ics@gencat.cat*

## Introduction

 Tooth brushing is a highly effective measure for preventing caries and periodontal diseases. Despite notable improvements in tooth brushing habits in Spain over recent years, there has been no corresponding reduction in gingival pathology. Daily tooth brushing does not always ensure effective biofilm control.

## Objectives

 To assess the oral hygiene level and gingival status of 1st and 6th grade primary school students in the La Marina de Port neighborhood, Barcelona.

## Methodology

 This cross-sectional descriptive study analyzed the results of school dental examinations conducted by two uncalibrated examiners between January and March 2023. Clinical variables included oral hygiene, categorized as "good" (minimal biofilm accumulation), "regular" (abundant and/or mature biofilm accumulation), or "poor" (generalized accumulation of mature and thick biofilm); and gingival status, classified as "healthy," "erythematous," or "calculus" based on clinical signs.

## Results

 A total of 171 schoolchildren were examined. Oral hygiene was classified as "good" in 43.8%, "regular" in 43.8%, and "poor" in 12.4%. For gingival status, 50% were "healthy," 42.7% were "erythematous," and 7.3% had "calculus." There was a significant decline in gingival health from 1st grade (82.8% "healthy") to 6th grade (25.5% "healthy").

## Conclusions

 Less than half of the students had good oral hygiene. Only half of the 1st graders and a quarter of the 6th graders showed no signs of gingival inflammation, underscoring the need for improved oral health education and preventive measures in this population.

# Abstract: Relationship between sleep disorders and dental occlusion in a school population

**Authors:** L. Messina, S. de Dios Teigell, P. Arrieta Blanco

**Affiliations:** *Faculty of Dentistry, Complutense University of Madrid (UCM), Spain. E-mail: lmessina@ucm.es*

## Introduction

 Literature suggests a relationship between malocclusions and sleep disorders.

## Objectives

 To evaluate the relationship between airway obstruction, sleep disorders, and dental occlusion in a school population from the Italian School of Madrid.

## Material and Methods

 A cross-sectional, prospective cohort study was conducted with 459 students aged 3-15 years from the Scuola Italiana di Madrid. The study was approved by the Ethics Committee of the Complutense University of Madrid. Students with current or previous orthodontic treatment were excluded. Variables included sleep disorders (BEARS test), transversal malocclusion (palatal compression), vertical malocclusion (biotype and overbite), anteroposterior malocclusion (Angle classification and facial profile), and soft tissue alterations (lip incompetence, oral breathing, extraoral muscle incompetence, Guilleminault scale, tonsil measurements, Friedman scale, modified Mallampati classification, and infantile swallowing). Two calibrated examiners performed measurements. Data were analyzed using SPSS version 10.0 and processed by the Statistics Department of the Complutense University of Madrid.

## Results

 The prevalence of sleep disorders (positive BEARS test) was 34.1%, considerably higher than in the general population (3.8%). Statistically significant differences were found in soft tissue variables: oral breathing (p<0.001), extraoral muscle hypotonia, and infantile swallowing (p<0.01) when comparing groups with and without sleep disorders. A higher tendency towards palatal compression was observed in patients with a positive BEARS test, although this was not statistically significant.

## Conclusions

 Students with a positive BEARS test significantly presented alterations in oral breathing, infantile swallowing, and muscle hypotonia. Early intervention in schools is crucial to prevent future problems.

# Abstract: The global burden of oral conditions

**Authors:** E. Bernabé

**Affiliations:** *Queen Mary University of London, United Kingdom. E-mail: e.bernabe@qmul.ac.uk*

## Introduction

 The presentation began by emphasizing that national and global estimates on the descriptive epidemiology of health conditions were crucial for policy planning and evaluation by governmental and non-governmental organizations. It was noted that the Global Burden of Disease (GBD) study utilized high-quality data to generate reliable estimates on the prevalence, incidence, and disability-adjusted life years (DALYs) of various diseases and injuries across different demographics and geographies.

## Objectives

 The aim of the presentation was to familiarize the audience with the methodology and findings of the global burden of oral conditions, focusing on untreated dental caries, severe periodontitis, and edentulism.

## Material and Methods

 The presentation was divided into two parts. The first part provided an overview of the GBD study methods, including data sources, data management, disease modeling, and burden estimation. The second part presented findings on (i) the prevalence and DALYs for each oral condition; (ii) trends in the burden of oral conditions from 1990 to 2021, analyzing the role of changes in population size, life expectancy, and disease rates; (iii) inequalities in disease burden according to the World Bank's classification of economies and WHO regions; and (iv) country-specific estimates for Spain relative to other European and OECD countries.

## Results

 The presentation revealed the current global burden of oral diseases and their trends over time. It highlighted significant disparities in oral health burdens across different economic classifications and geographic regions. Specific findings for Spain were contextualized within the broader European and OECD landscape.

## Conclusions

 The session concluded by emphasizing the challenges in addressing urgent oral health needs worldwide, particularly in low- and middle-income countries. The findings underscored the importance of using comprehensive, data-driven approaches to inform oral health policies and interventions globally.

# Abstract: Oral Health Data Bank Insights. A quick look at epidemiology

**Authors:** J.M. Montiel Company

**Affiliations:** *Department of Stomatology, University of Valencia, Spain. E-mail: jose.maria.montiel@uv.es*

## Introduction

 Oral Health Data Bank Insights (ohdbi) is an innovative and powerful visualizer of epidemiological data on oral health, encompassing over 40 indicators from 56 national and regional studies from 1969 to the present day. The project is being developed by José María Montiel-Company and Aarón W. Montiel Nicholas, creators of the Insights version.

## Objectives

 To show the main functions of the visualizer, introduce two new indicators and analyze the epidemiological trend of oral diseases in Spain.

## Material and Methods

 ohdbi is able to generate more than 20,000 different graphs due to its dynamism and user interaction. It includes the inter-community analyzer that incorporates an epidemiological trend generator that allows the visualization of the evolution over time of the main oral diseases, especially dental caries and periodontal disease, as well as a rankiometer.

## Results

 ohdbi proposes two new epidemiological indicators: a) Concentrated Caries Index (CCI), proposed by Montiel-Company JM and Iranzo-Cortés JE to assess the 80/20 phenomenon and the skewed distribution of caries. It is calculated by dividing the CAOD/cod index of the fifth part of the sample distribution with the highest CAOD/cod indices by the CAOD/cod index of the entire sample distribution. On a range scale between 1 and 5, if the result of the ratio is equal to 4, we are in an exact 80/20 phenomenon, i.e. 80% of the caries are concentrated in 20% of the population. If the result is greater than 4, there is an increase in the concentration of caries. On the contrary, if less than 4, a phenomenon of caries dispersion. b) Community Index of Dental and Periodontal Health (ICSDP), proposed by Montiel-Company JM, which, on a scale of 0 to 10, assesses the state of the dentition based on the cod/CAOD index and adds a correction based on the CPI of a population. It calculates a global index from the weighted average of the ICSDP of 5 cohorts studied, having assigned a percentage weight correlated with the population percentage of each cohort obtained from its distribution in the demographic pyramid.

## Conclusions

 Oral Health Data Bank Insights is a reference and pioneering tool in Spain with important applications in teaching, research and decision making in Oral Public Health, and available at https://www.uv.es/ohdbi/. 

# Abstract: Advances in Oral Microbiome Research

**Authors:** A. Mira

**Affiliations:** *FISABIO Foundation, Valencia, Spain. E-mail: alex.mira@fisabio.es*

## Introduction

 The oral microbiome plays a crucial role in maintaining oral and systemic health. Recent advances in microscopy, sequencing technologies and bioinformatics have revolutionized our understanding of the complex microbial communities inhabiting the oral cavity and their interactions with the host.

## Objectives

 To present recent advances in oral microbiome research, highlighting the journey from laboratory discoveries to clinical applications, with a focus on the identification of beneficial bacteria and the exploration of oral-systemic health connections.

## Material and Methods

 This presentation reviewed key findings from recent microbiome studies, including the speaker's work on *Streptococcus dentisani* and other potentially beneficial oral bacteria, like *Rothia* species which are able to reduce dietary nitrate. It discussed the application of multi-omics approaches in oral microbiome research and the development of novel prebiotic and probiotic strategies for oral health. The impact of new CLASI-FISH microscopy techniques was also discussed. The presentation also explored the emerging evidence linking oral microbiome dysbiosis to systemic diseases.

## Results

 The identification of * S. dentisani *and other health-associated bacteria has opened new avenues for probiotic interventions in oral health. Metagenomic and metatranscriptomic analyses have revealed complex interactions within oral microbial communities and between microbes and the host. These findings are informing the development of targeted therapies and diagnostic tools and prevention. Furthermore, associations between oral microbiome composition and systemic conditions such as cardiovascular disease, diabetes, and cancer have been identified, suggesting potential for microbiome-based biomarkers and therapeutic targets.

## Conclusions

 Advances in oral microbiome research are transforming our approach to oral health management and offering new insights into the oral-systemic health axis. The transition from laboratory discoveries to clinical applications holds promise for personalized, microbiome-based strategies in dentistry and medicine. However, further research is needed to fully elucidate the mechanisms underlying microbiome-host interactions and to translate these findings into effective clinical interventions.

# Abstract: Diagnosis of Initial Caries Lesions: Talent and Technology

**Authors:** R. Garcillán

**Affiliations:** *Complutense University of Madrid, Spain. E-mail: mrgarcil@odon.ucm.es*

## Introduction

 The presentation began by emphasizing that effective caries control required a risk-based diagnostic approach and an individualized treatment plan. It was noted that the CAMBRA protocol allowed for patient-specific risk assessment, aiming to shift the balance towards oral health. While traditional diagnostic methods such as visual-tactile inspection and radiological examination remained crucial, it was highlighted that new non-invasive technologies had emerged to enhance early caries detection.

## Objectives

 The aim of the presentation was to review current diagnostic methods for initial caries lesions, emphasizing the combination of clinical expertise and technological advancements for improved caries management.

## Material and Methods

 The presentation explored various diagnostic approaches, including visual-tactile examination using the ICDAS II classification, radiographic methods, and fluorescence-based technologies such as the DIAGNOdent Pen. The efficacy of these methods was discussed, along with their limitations and optimal applications. Additionally, the use of magnification devices in dentistry was examined for their potential to enhance diagnostic accuracy and improve ergonomics.

## Results

 The presentation revealed that the highest sensitivity for caries diagnosis was achieved through visual and tactile assessment using the ICDAS II classification, combined with laser fluorescence from the DIAGNOdent Pen. It was noted that radiographic methods remained valuable, particularly for interproximal lesions, though they were limited in detecting early-stage caries. The discussion also highlighted that magnification tools in dentistry offered improved visualization of the oral cavity, potentially leading to better diagnoses and enhanced ergonomics for dental professionals.

## Conclusions

 The presentation concluded that a multifaceted approach combining clinical expertise with advanced diagnostic technologies was essential for effective early caries detection and management. It was emphasized that while technological aids enhanced diagnostic capabilities, they should complement rather than replace thorough clinical examination. The need for further research was highlighted to fully understand the adaptation of dental professionals to new diagnostic devices and their long-term impact on caries management outcomes.

# Abstract: Teledentistry: A Public Oral Health Tool

**Authors:** N. Giraudeau

**Affiliations:** *University of Montpellier, France. E-mail: nicolas.giraudeau@umontpellier.fr*

## Introduction

 The presentation began by introducing the World Health Organization's definition of teledentistry as the "use of ICT for dental consultations, diagnosis and treatment planning, including the transmission of clinical information and images between an oral health professional and patient or between two health professionals, including at least one oral health professional, who are separated by distance." It was noted that despite the increasing implementation of teledentistry projects worldwide, numerous questions remained unanswered, with the most crucial being: "Why should we use teledentistry?"

## Objectives

 The aim of the presentation was to examine the rationale behind teledentistry implementation and explore its potential as a public health tool, with a focus on its role in reducing oral health inequalities.

## Material and Methods

 The presentation critically analyzed the approach to teledentistry use and implementation from a public health perspective. It explored the fundamental principles that should guide teledentistry adoption, emphasizing its potential to decrease rather than exacerbate health inequalities.

## Results

 The discussion highlighted two fundamental points: the importance of adopting a public health approach in teledentistry implementation, and the critical need for teledentistry to serve as a tool for reducing oral health disparities rather than widening them. Case studies and current research were presented to illustrate these points.

## Conclusions

 The presentation concluded that teledentistry held significant promise as a public oral health tool, but its implementation needed to be guided by clear public health principles. It was emphasized that the technology should be leveraged to address existing oral health inequalities and improve access to care for underserved populations. The presentation suggested that future research and policy development in teledentistry should prioritize its potential to create more equitable oral health outcomes across diverse communities.

# Abstract: Role of Dental Hygienists as Part of the Team Necessary for the Development of the WHO Global Oral Health Strategy

**Authors:** E. López de Castro

**Affiliations:** *Spanish Federation of Dental Hygienists (HIDES), Spain. E-mail: evalopezdcastro@hotmail.com*

## Introduction

 The presentation began by discussing the World Health Organization's (WHO) Global Oral Health Action Plan (OHAP) 2023-2030, which had been finalized after public consultation in 2022 and was now on the global policy agenda. It was emphasized that with oral diseases being the most prevalent non-communicable diseases (NCDs), it was crucial for dental hygienists, as members of oral health teams and experts in prevention and health promotion, to be aware of and engaged with the Global Action Plan for NCD prevention and control.

## Objectives

 The aim of the presentation was to examine the role of dental hygienists in implementing the WHO's Global Oral Health Action Plan, highlighting the opportunities and challenges in the Spanish context.

## Material and Methods

 The presentation analyzed the OHAP 2023-2030 and its implications for dental hygienists. It also reviewed the current status of dental hygienists in Spain's oral health teams, comparing it with other European countries. The legal framework supporting the professional competencies of dental hygienists in Spain was assessed.

## Results

 The presentation demonstrated that the OHAP 2023-2030 represented a significant opportunity for dental hygienists to expand their role in oral health promotion and disease prevention. However, the analysis revealed disparities in the integration of dental hygienists into oral health teams across different autonomous communities in Spain. These disparities, along with differences in professional standing compared to other European countries, highlighted the need for a more standardized approach to dental hygienists' roles and responsibilities.

## Conclusions

 The presentation concluded that to effectively contribute to the WHO's Global Oral Health Strategy, there was a need for greater homogenization of dental hygienists' roles across Spain's autonomous communities. It was suggested that legal tools supporting the professional competencies of dental hygienists should be reassessed and potentially strengthened. The presentation emphasized that this would enable dental hygienists to fully leverage their expertise in prevention and health promotion, aligning with global oral health objectives and improving oral health outcomes in Spain.

# Abstract: Contemporary dental caries management using the minimum intervention oral care delivery framework

**Authors:** A. Banerjee

**Affiliations:** *Faculty of Dentistry, Oral & Craniofacial Sciences, King's College London, United Kingdom. E-mail: avijit.banerjee@kcl.ac.uk*

## Introduction

 The presentation began by highlighting how the management of dental caries had evolved significantly in recent years, moving towards a more person-centered, preventive approach. It was explained that the minimum intervention oral care (MIOC) delivery framework represented a modern, team-delivered strategy for managing dental caries in all patients.

## Objectives

 The aim of the presentation was to outline the MIOC delivery framework and its four interlinking clinical domains for comprehensive caries management. Additionally, it sought to introduce postgraduate educational opportunities in this field.

## Material and Methods

 The presentation described the MIOC framework, focusing on its four key clinical domains. These included identifying disease, which encompassed detection, diagnosis, risk/susceptibility assessment, and personalized care planning. The second domain, prevention of lesions / control of disease, covered primary non-operative and secondary non-operative / micro-invasive interventions, as well as person behavior management. The third domain, minimally invasive operative interventions (MID), addressed tertiary prevention of cavitated deeper lesions. Finally, the fourth domain, re-assessment and active surveillance, included longitudinal risk/susceptibility assessment.

## Results

 The presentation demonstrated that the MIOC framework provided a comprehensive, risk-related, and prevention-based approach to dental caries management. It emphasized the importance of early detection, personalized risk assessment, and minimally invasive interventions. This approach was shown to align with contemporary understanding of caries as a dynamic disease process and focused on preserving tooth structure while managing the disease effectively.

## Conclusions

 The presentation concluded that the MIOC delivery framework represented a paradigm shift in dental caries management, moving away from traditional "drill and fill" approaches towards a more holistic, preventive strategy. It was emphasized that its implementation required a team-based approach and continuous education. To support this, the presentation also highlighted postgraduate educational opportunities, specifically the blended-learning masters course in Advanced Minimum Intervention Restorative Dentistry (AMIRD) offered at King's College London for practicing dentists and dental therapists.

# Abstract: Childsmile: The Scottish child caries prevention programme

**Authors:** C. Jones

**Affiliations:** *University of Edinburgh, United Kingdom. E-mail: Colwyn.Jones@glasgow.ac.uk*

## Introduction

 The presentation began by describing Childsmile as a government-funded programme designed to improve child dental health in Scotland and reduce societal dental health inequalities. It was explained that the programme, implemented in 2011, combined evidence-based approaches that had led to steady improvements in the dental health of Scottish children.

## Objectives

 The aim of the presentation was to present the structure, implementation, and outcomes of the Childsmile programme, including its impact on reducing tooth decay and socioeconomic inequalities in dental health among Scottish children. Additionally, it sought to discuss the potential effects of the SARS-CoV-2 pandemic on the programme.

## Material and Methods

 The presentation outlined the key components of the Childsmile programme. These included early intervention, where health visitors introduced Childsmile to new parents, providing oral health advice and free toothbrushes and fluoride toothpaste from 6 months of age. It also described nursery education, where all Scottish children received 30 hours per week of free nursery education from ages 3 to 5, including daily toothbrushing practice and healthy diet promotion. Targeted interventions were explained, where children in areas of relative socio-economic deprivation received 6-monthly topical fluoride varnish applications in nursery and the first two years of primary school. Finally, dental practice involvement was detailed, with routine 6-monthly check-ups including topical fluoride application, and ongoing preventive care provided after age 7.

## Results

 The presentation showed that the Childsmile programme had successfully reduced levels of tooth decay in 5 and 11-year-old children. Progress on reducing socioeconomic inequalities in dental health was presented. The presentation also covered the possible effects of the SARS-CoV-2 global pandemic on the Childsmile programme and its outcomes.

## Conclusions

 The presentation concluded that Childsmile represented a comprehensive, evidence-based approach to improving child dental health and reducing health inequalities. It was highlighted that its multi-faceted strategy, involving various stakeholders from health visitors to dental practitioners, had shown promising results in reducing tooth decay among Scottish children. However, it was emphasized that the impact of the recent pandemic on the programme's implementation and effectiveness needed careful consideration. It was suggested that this model of early intervention and targeted prevention could provide valuable insights for other regions seeking to improve child dental health and reduce oral health inequalities.

# Abstract: The role of community nursing in improving oral health

**Authors:** L. Almudéver Campo

**Affiliations:** *Official College of Nursing of Valencia, Spain. E-mail: laura.almudever@uv.es *

## Introduction

 The presentation began by discussing the World Health Organization's (WHO) global strategy on oral health, which is based on the United Nations 2030 Agenda for Sustainable Development, particularly Goal 3 (Ensure healthy lives and promote well-being for all at all ages) and its target 3.8, aimed at achieving universal health coverage. Oral health was defined as the health of the mouth and teeth that enables people to perform basic functions and affects psychosocial dimensions of well-being.

## Objectives

 The presentation aimed to highlight the fundamental role of nurses in developing and implementing oral health programs, preventing risk factors, and early detection of non-communicable diseases (NCDs). Additionally, it sought to emphasize the importance of Health Education programs in promoting lifestyle changes and oral cavity care.

## Material and Methods

 The presentation explored the association between oral diseases and other NCDs, particularly periodontal diseases with diabetes and cardiovascular diseases. It also examined the role of nurses in oral health promotion and disease prevention, as well as the effectiveness of Health Education programs aimed at lifestyle changes and oral care. Strategies for nurses to improve oral health across populations and prevent future complications were also discussed.

## Results

 The presentation demonstrated that nurses play a crucial role in delivering quality care to a broader population through their involvement in oral health programs and NCD prevention. It was shown that Health Education programs focused on lifestyle changes and oral care have demonstrated effectiveness. The presentation highlighted how nurses can leverage these programs to improve oral health outcomes and prevent future complications.

## Conclusions

 The presentation concluded that community nursing has a significant impact on improving oral health through education, prevention, and early intervention. By focusing on the promotion and dissemination of effective Health Education programs, it was emphasized that nurses can contribute to enhancing the oral health of entire populations. This approach was shown to align with the WHO's global strategy on oral health and support the achievement of universal health coverage. The interdisciplinary nature of oral health care, particularly its connection with other NCDs, was underscored as highlighting the importance of nurses in comprehensive healthcare delivery.

# Abstract: Anticoagulants and Antiplatelet Agents in Dentistry: How to Proceed?

**Authors:** V. Bertomeu Martínez

**Affiliations:** *International Institute of Cardiology, Catholic University of San Antonio of Murcia (UCAM), Spain. E-mail: bertomeu.consulta@gmail.com*

## Introduction

 Anticoagulants and antiplatelet agents are commonly used medications that can significantly impact dental procedures. While general guidelines exist, it's crucial to recognize that each patient requires individualized treatment based on their specific circumstances. Understanding the indication for anticoagulation is vital to assess the risk of ischemic events, while the bleeding risk depends on the type of dental procedure.

## Objectives

 The presentation aimed to provide an overview of how to manage patients on anticoagulant and antiplatelet therapy in dental settings, emphasizing the importance of individualized assessment and interdisciplinary communication.

## Material and Methods

 The presentation covered oral anticoagulants, including Vitamin K antagonists (e.g., Warfarin, Acenocoumarol) and Direct-Acting Oral Anticoagulants (DOACs), as well as antiplatelet agents such as Aspirin and other drugs (e.g., clopidogrel, prasugrel, ticagrelor). It discussed monitoring INR levels for Vitamin K antagonists, understanding the more stable nature of DOACs, guidelines for low-dose and high-dose aspirin usage, and considerations for other antiplatelet drugs in routine and major dental procedures. The importance of interdisciplinary communication between dentists and physicians in decision-making was also emphasized.

## Results

 The presentation highlighted that while general guidelines exist, each patient's case must be evaluated individually. For vitamin K antagonists, INR monitoring was emphasized as crucial. DOACs were noted to require different considerations due to their pharmacological properties. It was explained that low-dose aspirin usually doesn't require discontinuation for routine procedures, but higher doses may need reassessment. Other antiplatelet drugs were generally not recommended to be stopped for routine procedures but may require evaluation for major surgeries.

## Conclusions

 The presentation concluded that managing patients on anticoagulant and antiplatelet therapy in dental settings requires a careful, individualized approach. The key to safe and effective treatment lies in understanding the specific medications, their indications, and potential risks. Close collaboration between dentists and the patient's physician was emphasized as essential to make informed decisions that ensure patient safety and optimal care. This approach allows for balancing the risk of thrombotic events against the risk of bleeding complications during dental procedures.

# Abstract: Workshop on materials for minimally invasive dentistry

**Authors:** Y. Martínez Beneyto

**Affiliations:** *Department of Preventive Dentistry, University of Murcia, Spain. E-mail: yolandam@um.es*

## Introduction

 Minimal intervention dentistry focuses on preserving natural dental tissues, reducing the need for invasive techniques. This approach requires the use of modern materials that allow for effective restorations and treatments with minimal intervention on teeth and gums.

## Objectives

 To provide an in-depth understanding of advanced materials for minimally invasive dentistry and their proper application techniques.

## Material and Methods

 The workshop covers advanced biomaterials, glass ionomers, and regenerative materials that promote natural tissue repair. Through practical demonstrations and theoretical discussions, participants learn to select appropriate materials based on biocompatibility, durability, strength, and aesthetics. The importance of accurate diagnosis and personalized treatment planning is emphasized.

## Results

 Participants gain knowledge on a) Characteristics and applications of modern minimally invasive dental materials; b) Proper handling techniques for these materials; c) Factors influencing material selection for specific clinical scenarios; d) Integration of minimally invasive approaches in comprehensive treatment planning.

## Conclusions

 This workshop equips dental professionals with the knowledge and skills to implement minimally invasive techniques effectively, promoting long-term dental health with a conservative approach that benefits both the functionality and aesthetics of patients.

# Abstract: Vital Pulp Therapy and Endodontics with Bioceramic Materials

**Authors:** J. Ghilotti

**Affiliations:** *University of Valencia, Spain. E-mail: gja@alumni.uv.es*

## Introduction

 Bioceramic materials have emerged as innovative solutions in dental procedures, particularly in vital pulp therapy and endodontics. These materials offer biocompatibility and sealing properties that can enhance treatment outcomes. However, their effective use requires specific knowledge and hands-on experience.

## Objectives

 The workshop aimed to provide participants with theoretical knowledge and practical skills in the application of bioceramic materials for vital pulp therapy and endodontic treatments. It was designed to familiarize dental professionals with the properties, indications, and handling techniques of these materials.

## Material and Methods

 The workshop was structured in two main parts: a theoretical introduction and practical sections. The theoretical part provided an overview of bioceramic materials, their clinical applications, and various formats for specific uses. The practical section was divided into two segments: vital pulp therapy and endodontic treatment. In the vital pulp therapy segment, participants gained hands-on experience with Biodentine for direct and indirect pulp capping on extracted teeth with deep caries. The endodontic treatment segment demonstrated root canal treatment using the Reciproc Blue single-file system and obturation technique using BioRoot RCS bioceramic cement, with practical exercises on extracted teeth with prepared access cavities.

## Results

 Participants gained a comprehensive understanding of bioceramic materials' properties and applications, practical skills in applying Biodentine for vital pulp therapy, experience in using the Reciproc Blue system for canal instrumentation, and proficiency in cold obturation technique using BioRoot RCS and gutta-percha.

## Conclusions

 This hands-on workshop provided dental professionals with valuable experience in using state-of-the-art bioceramic materials for vital pulp therapy and endodontic treatments. By combining theoretical knowledge with practical application, participants are now better equipped to incorporate these advanced materials into their clinical practice, potentially improving treatment outcomes and patient care.

# Abstract: Clinical Caries Diagnosis Workshop Using the ICDAS System

**Authors:** C. Llena

**Affiliations:** *Department of Stomatology, University of Valencia, Spain. E-mail: llena@uv.es*

## Introduction

 The International Caries Detection and Assessment System (ICDAS II) is a consensus-based system for caries detection and diagnosis, designed for clinical practice, research, and public health. This system aims to provide a standardized visual method for detecting caries in its earliest stages and assessing its severity and activity level.

## Objectives

 To familiarize participants with the ICDAS II coding system and train them in the clinical diagnosis of caries lesions with varying extents and activity levels.

## Material and Methods

 The workshop was structured in two parts. First, a comprehensive review of the ICDAS II coding system was presented. The system's two-digit nomenclature was explained, with the first digit (0-8) corresponding to restoration and sealant codes, and 9 indicating a missing tooth. The second digit, comprising seven categories (0-6), corresponds to the clinical diagnosis of caries, ranging from healthy tooth (0) to exposed dentine caries (5-6). The workshop then provided hands-on training in clinical diagnosis using this system. Additionally, the International Caries Classification and Management System (ICCMS), which builds upon ICDAS II, was introduced as a standardized method for integrating and synthesizing tooth and patient information, including caries risk status, for comprehensive caries management in clinical practice.

## Results

 Participants gained practical experience in applying the ICDAS II codes to various caries presentations. They learned to distinguish between different stages of caries progression, from early enamel changes to dentine involvement. The workshop also demonstrated how the ICDAS II system interfaces with the ICCMS, providing a framework for risk-based caries management and monitoring in clinical settings.

## Conclusions

 The workshop provided dental professionals with enhanced skills in standardized caries detection and diagnosis using the ICDAS II system. By integrating this system with the ICCMS approach, participants were equipped with tools for more comprehensive, patient-centered caries management. This standardized approach to caries diagnosis and management has the potential to improve both clinical practice and public health interventions in dentistry.
